# Trends in Neurotrauma Epidemiology, Management, and Outcomes during the COVID-19 Pandemic in Kigali, Rwanda

**DOI:** 10.1089/neu.2022.0166

**Published:** 2023-03-01

**Authors:** Oliver Y. Tang, Chantal Uwamahoro, Catalina González Marqués, Aly Beeman, Enyonam Odoom, Vincent Ndebwanimana, Doris Uwamahoro, Mediatrice Niyonsaba, Apollinaire Nzabahimana, Silas Munyanziza, Steven Nshuti, Spandana Jarmale, Andrew H. Stephen, Adam R. Aluisio

**Affiliations:** ^1^ Division of Global Emergency Medicine, Warren Alpert Medical School of Brown University, Providence, Rhode Island, USA.; ^2^ Department of Anesthesia, Emergency Medicine, and Critical Care, University of Rwanda, Kigali, Rwanda.; ^3^ Department of Emergency Medicine, Brigham and Women's Hospital, Boston, Massachusetts, USA.; ^4^ Department of Emergency Medicine, Warren Alpert Medical School of Brown University, Providence, Rhode Island, USA.; ^5^ Department of Neurosurgery, King Faisal Hospital, Kigali, Rwanda.

**Keywords:** COVID-19, global health, low- and middle-income countries, neurosurgery, neurotrauma, spine trauma, Sub-Saharan Africa, traumatic brain injury

## Abstract

National regulations to curb the coronavirus disease 2019 (COVID-19) transmission and health care resource reallocation may have impacted incidence and treatment for neurotrauma, including traumatic brain injury (TBI) and spinal trauma, but these trends have not been characterized in Sub-Saharan Africa. This study analyzes differences in epidemiology, management, and outcomes preceding and during the COVID-19 pandemic for neurotrauma patients in a Rwandan tertiary hospital. The study setting was the Centre Hospitalier Universitaire de Kigali (CHUK), Rwanda's national referral hospital. Adult injury patients presenting to the CHUK Emergency Department (ED) were prospectively enrolled from January 27, 2020 to June 28, 2020. Study personnel collected data on demographics, injury characteristics, serial neurological examinations, treatment, and outcomes. Differences in patients before (January 27, 2020 to March 21, 2020) and during (June 1, 2020 to June 28, 2020) the COVID-19 pandemic were assessed using chi-squared and Mann-Whitney *U* tests. The study population included 216 patients with neurotrauma (83.8% TBI, 8.3% spine trauma, and 7.9% with both). Mean age was 34.1 years (standard deviation [SD] = 12.5) and 77.8% were male. Patients predominantly experienced injury following a road traffic accident (RTA; 65.7%). Weekly volume for TBI (mean = 16.5 vs. 17.1, *p* = 0.819) and spine trauma (mean = 2.0 vs. 3.4, *p* = 0.086) was similar between study periods. During the pandemic, patients had lower Glasgow Coma Scale (GCS) scores (mean = 13.8 vs. 14.3, *p* = 0.068) and Kampala Trauma Scores (KTS; mean = 14.0 vs. 14.3, *p* = 0.097) on arrival, denoting higher injury severity, but these differences only approached significance. Patients treated during the pandemic period had higher occurrence of hemorrhage, contusion, or fracture on computed tomography (CT) imaging (47.1% vs. 26.7%, *p* = 0.003) and neurological decline (18.6% vs. 7.5%, *p* = 0.016). Hospitalizations also increased significantly during COVID-19 (54.6% vs. 39.9%, *p* = 0.048). Craniotomy rates doubled during the pandemic period (25.7% vs. 13.7%, *p* = 0.003), but mortality was unchanged (5.5% vs. 5.7%, *p* = 0.944). Neurotrauma volume remained unchanged at CHUK during the COVID-19 pandemic, but presenting patients had higher injury acuity and craniotomy rates. These findings may inform care during pandemic conditions in Rwanda and similar settings.

## Introduction

Low- and middle-income countries (LMICs) disproportionately experience the burden of injuries, which account for approximately 90% of global deaths and disability-adjusted life years (DALYs) lost due to trauma.^1,2^ Among different injury mechanisms, central nervous system (CNS) traumatic insults to the brain or spine are associated with the greatest mortality and morbidity, including over 8 million DALYs lost annually.^3,4^ An estimated 27–69 million individuals experience traumatic brain injury (TBI) annually, with a threefold higher burden of injury in LMICs.^3,5^ Moreover, patients with TBI in LMICs are twice as likely to die than those in high-income countries (HICs).^6^ In Kigali, the capital of Rwanda, CNS trauma is responsible for two-thirds of mortalities responded to by pre-hospital emergency medical services.^7^

The coronavirus disease 2019 (COVID-19) pandemic, caused by the severe acute respiratory syndrome coronavirus 2 (SARS-CoV-2), has created challenges in the management of injury patients requiring emergency care globally. Several studies in the United States have documented significant decreases in caseloads for brain and spine trauma as well as other emergent conditions, such as stroke and myocardial infarction.^8–11^ Despite this decreased volume during the COVID-19 pandemic, higher severity of presentation and mortality have been documented for these conditions, which studies have partially attributed to decreased and delayed presentation for acute care.^8,12,13^ Additional studies documenting similar trends in other HICs have documented COVID-19's indirect impact on the treatment of trauma due to disruptions of normal care processes, staffing, and supplies.^14–17^ Although these trends have been evaluated in HICs, potential disruptions in trauma care in LMICs have been minimally characterized. Some studies have analyzed changes in general trauma within Africa,^18–22^ but minimal CNS-trauma-specific assessments have been undertaken to date.

The high burden of CNS trauma in LMICs is additionally complicated by reduced baseline resources for injury management. During the COVID-19 pandemic, given changes in health care resource allocation and nationwide policies to promote public health, such as restrictions in patient mobility, it is conceivable that neurotrauma patient care was impacted by the pandemic. Rwanda, recognized for having one of the most successful pandemic responses in Africa, notably executed several pandemic-related changes during early 2020, including the first national lockdown implemented on the continent.^23^ Accordingly, this study aimed to evaluate differences in presenting characteristics, management, and outcomes for patients with CNS trauma before and during the COVID-19 pandemic at Rwanda's primary national public referral hospital.

## Methods

### Study setting and population

This prospective cross-sectional study was conducted at the Centre Hospitalier Universitaire de Kigali (CHUK) in Kigali, Rwanda.^22^ CHUK is Rwanda's primary national referral hospital, with approximately 500 inpatient beds and a 50-bed emergency department (ED) providing 24-h daily care, and the only neurosurgery service supported by the Rwanda Ministry of Health nationwide.^24,25^

**FIG. 1.**
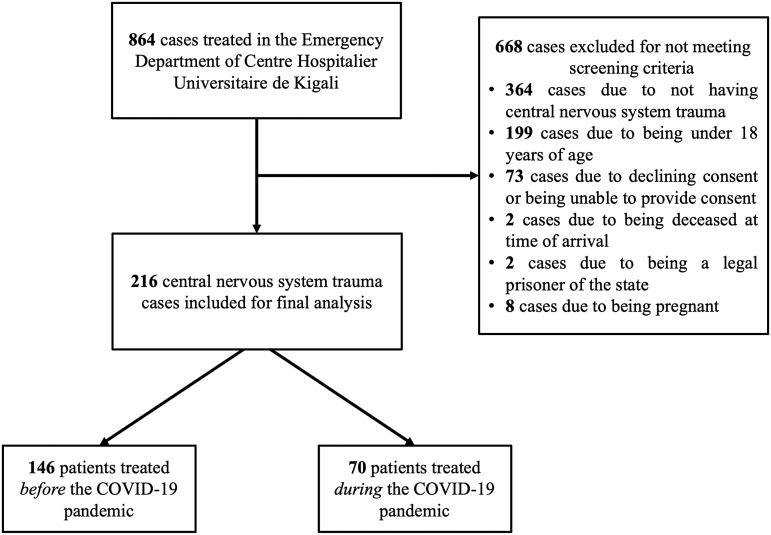
Flow diagram for CNS trauma patients at CHUK. The diagram demonstrates application of inclusion and exclusion criteria for the present study. A total of 216 CNS trauma patients treated at CHUK were identified for analysis. CHUK, Centre Hospitalier Universitaire de Kigali; CNS, central nervous system; COVID-19, coronavirus disease 2019.

Under the provisions of an Institutional Review Board-approved protocol developed before the COVID-19 pandemic focused on general trauma epidemiology at CHUK, all adult injury patients presenting to the CHUK ED were screened for enrollment. Adult injury patients presenting with neurotrauma who were not deceased on arrival, a prisoner of the state, or pregnant and who provided consent were included for analysis. A legally authorized patient representative provided informed consent for patients unable to consent due to incapacity. The present CNS-trauma-focused analysis is a subpopulation analysis of a previously published study for the general trauma population at CHUK, as described earlier, which did not analyze the CNS trauma in isolation.^22^ Patients were enrolled from January 27, 2020 to June 28, 2020 excluding a cessation of research activities from March 22, 2020 to May 31, 2020 during Rwanda's first national lockdown.^22^ Consequently, the study population was stratified by date of presentation into groups: “before COVID-19” (January 27, 2020 to March 21, 2020) and “during COVID-19” (June 1, 2020 to June 28, 2020). Exclusion criteria are visualized in Figure 1, with the most common reasons for exclusion being not having CNS trauma (*n* = 364) and being <18 years of age (*n* = 199). All research activities were approved by the CHUK Research Ethics Committee (EC/CHUK/053/2021) and Institutional Review Board of Brown University (#21-32).

### Data management

Following patient enrollment, trained research personnel collected data continuously using structured case reporting forms on an hourly basis during the first 6 h of ED care. Variables prospectively collected included patient demographics, mechanism of injury, time from injury to presentation, injury acuity, acute interventions during the first 6 h of care, imaging findings, and disposition. Injury severity was quantified by the Glasgow Coma Scale (GCS) and Kampala Trauma Score (KTS). The KTS is a previously validated trauma prognostication score designed specifically for use in LMIC and ranges continuously from 5 to 16, with lower scores denoting higher acuity.^26–28^ Following earlier studies, the shock index was calculated as the quotient of heart rate over systolic blood pressure on ED arrival and dichotomized at a threshold of ≥0.9 to indicate hemodynamic instability.^29^

To identify additional clinical variables of interest not captured by standardized screening forms, a retrospective chart review was conducted to record symptoms on presentation, serial neurological examinations, neurological intensive care unit (neuro-ICU) admission and length of stay (LOS), pharmacological agents administered, overall LOS, and extended Glasgow Outcome Scale (GOSE) score at discharge, reflecting functional status.^30,31^ GCS score (mild = 13–15, moderate = 9–12, severe = 3–8) and GOSE score (7–8 = good recovery vs. 1–6 = death, vegetative state, or disability) were categorized following earlier methods.^28,32^ The outcome of neurological decline was defined as a composite variable encapsulating GCS score decline of ≥2 points, new-onset unconsciousness, or new-onset sensorimotor deficit documented on serial exams.^33,34^

Study enrollment and standardized data collection were performed by a team of seven research personnel who remained constant through the study period, all native to Rwanda, fluent in the three local languages (Kinyarwanda, French, and English), and with experience in clinical research and health care provision. Research personnel were trained in operating procedures by via didactic sessions and interactive role playing over a 2-day seminar. Data were collected on standardized hard-copy case reporting forms and entered daily into a password-protected online database. Data were checked weekly by the study team for validation and any data collection personnel having >5% missingness in their data were given focused assistance and retraining to improve data capture.

### Statistical analysis

All statistical analysis was performed via Stata 17 (StataCorp, College Station, TX, USA). Data visualization of end-points of clinical interest chosen *a priori* was conducted using the *matplotlib* package on Python (Centrum voor Wiskunde en Informatica Amsterdam, The Netherlands). Descriptive statistics calculated for the study population included frequencies with percentages for categorical variables and means with standard deviations (SDs) or medians with interquartile ranges (IQRs) for continuous variables. Differences in patient ED volume, presenting characteristics, management, and outcomes for patients treated before compared with during the COVID-19 pandemic were queried by chi-squared or Fisher's exact tests for categorical variables and the non-parametric Mann-Whitney *U* test for continuous variables. Missing data that were not collected prospectively via standardized study forms or could not be abstracted from chart review were included as a discrete category during analysis of categorical variables. Statistical significance was maintained at *p* < 0.05, without correction for multiple comparisons.

## Results

### Characteristics of study population

Among 864 injury cases treated at the CHUK ED during the study period, 216 adult patients with neurotrauma were analyzed (Fig. 1), including 83.8% with TBI, 8.3% with spine trauma, and 7.9% with both TBI and spine trauma (Table 1). Mean patient age was 34.1 years (SD = 12.5 years) and most patients were male (77.8%). The most common mechanisms of injury were RTA (65.7%), blunt assault (10.6%), and fall (8.8%). Thirteen percent of patients had documented alcohol use before injury. The median time from injury to ED presentation was 3 h (IQR = 1–14 h). Additionally, 41.2% and 31.9% of patients were transported by pre-hospital services and transferred from another facility, respectively.

**Table 1.** Characteristics of CNS Trauma Patients Treated before and during the COVID-19 Pandemic

Variable	All patients (*n* = 216)	Treated before COVID-19 (*n* = 146)	Treated during COVID-19 (*n* = 70)	*P*-value
Type of CNS trauma Traumatic brain injury Spine injury Both	181 (83.8%) 18 (8.3%) 17 (7.9%)	119 (81.5%) 14 (9.6%) 13 (8.9%)	62 (88.6%) 4 (5.7%) 4 (5.7%)	0.418
Age: Median (IQR)	31 (25–40)	30 (25–38)	33 (26–42)	0.144
Sex Male Female	168 (77.8%) 48 (22.2%)	39 (26.7%) 109 (73.3%)	9 (12.9%) 61 (87.1%)	0.022
Mechanism of injury^a^ Road traffic accident Blunt assault Fall Stab or cut Other blunt injury Other penetrating injury	142 (65.7%) 23 (10.6%) 19 (8.8%) 14 (6.5%) 16 (7.4%) 2 (0.9%)	97 (66.4%) 14 (9.6%) 11 (7.5%) 11 (7.5%) 10 (6.9%) 2 (1.4%)	45 (64.3%) 9 (12.9%) 8 (11.4%) 3 (4.3%) 6 (8.6%) 0 (0.0%)	0.755 0.466 0.344 0.364 0.651 0.325
Alcohol use before injury Yes No Missing	28 (13.0%) 65 (30.1%) 123 (56.9%)	16 (11.0%) 49 (33.6%) 81 (55.5%)	12 (17.1%) 16 (22.9%) 42 (60.0%)	0.186
Hours between injury and presentation: Median (IQR)	3 (1–14)	3 (1–12)	3 (1–20)	0.877
Patient transported by pre-hospital services	69 (31.9%)	47 (32.2%)	22 (31.4%)	0.607
Patient transferred from another health facility	89 (41.2%)	55 (37.7%)	34 (48.6%)	0.128
Presence of polytrauma	119 (55.1%)	85 (58.2%)	34 (48.6%)	0.182
Type of injury Blunt Penetrating	200 (92.6%) 16 (7.4%)	133 (91.1%) 13 (8.9%)	67 (95.7%) 3 (4.3%)	0.225
Shock Index <0.9 ≥0.9	196 (90.7%) 20 (9.3%)	132 (90.4%) 14 (9.6%)	64 (91.4%) 6 (8.6%)	0.809
GCS: Median (IQR) 13–15 9–12 3–8	15 (15–15) 187 (86.6%) 19 (8.8%) 10 (4.6%)	15 (15–15) 130 (89.0%) 11 (7.5%) 5 (3.4%)	15 (14.5–15) 57 (81.4%) 8 (11.4%) 5 (7.1%)	0.068 0.280
Kampala Trauma Score: Median (IQR)	14 (14–15)	15 (14–15)	14 (14–15)	0.097
Consciousness on ED arrival Conscious Unconscious	159 (73.6%) 57 (26.4%)	111 (76.0%) 35 (24.0%)	48 (68.6%) 22 (31.4%)	0.507
AVPU Scale on ED arrival Alert Verbal Pain Unresponsive	163 (75.5%) 14 (6.5%) 31 (14.4%) 8 (3.7%)	115 (78.8%) 7 (4.8%) 20 (13.7%) 4 (2.7%)	48 (68.6%) 17 (10.0%) 11 (15.7%) 4 (5.7%)	0.273
Mobility on ED arrival Walking independently Walking with help Stretcher or immobile Missing	37 (17.1%) 52 (24.1%) 108 (50.0%) 19 (8.8%)	37 (25.3%) 28 (19.2%) 71 (48.6%) 10 (6.9%)	15 (21.4%) 9 (12.9%) 37 (52.9%) 9 (12.9%)	0.319
Sensorimotor deficits on ED arrival No deficits Motor deficit Sensory deficit Motor and sensory deficit Missing	154 (71.3%) 4 (1.9%) 10 (4.6%) 27 (12.5%) 21 (9.7%)	111 (76.0%) 3 (2.1%) 6 (4.1%) 15 (10.3%) 11 (7.5%)	43 (61.4%) 1 (1.4%) 4 (5.7%) 12 (17.1%) 10 (14.3%)	0.216
Spinal cord injury in spine trauma patients (*n* = 35) No spinal cord injury Incomplete Complete	15 (42.9%) 15 (42.9%) 5 (14.3%)	11 (40.7%) 13 (48.2%) 3 (11.1%)	4 (50.0%) 2 (25.0%) 2 (25.0%)	0.421
Neurological decline during admission	24 (11.1%)	11 (7.5%)	13 (18.6%)	0.016
Trauma treatment^a^ Surgery consultation Craniotomy or craniectomy Spine immobilization Intubation Antibiotic administration Antiepileptic drug administration Stress ulcer prophylaxis	117 (54.2%) 38 (17.6%) 28 (13.0%) 32 (14.8%) 121 (56.0%) 40 (18.5%) 21 (9.7%)	73 (50.0%) 20 (13.7%) 18 (12.3%) 20 (13.7%) 82 (56.2%) 24 (16.4%) 16 (11.0%)	44 (62.9%) 18 (25.7%) 10 (14.3%) 12 (17.1%) 39 (55.7%) 16 (22.9%) 5 (7.1%)	0.076 0.030 0.689 0.505 0.950 0.256 0.376
CT imaging performed	136 (63.0%)	87 (60.0%)	49 (70.0%)	0.138
Abnormal CT findings documented	72 (33.3%)	39 (26.7%)	33 (47.1%)	0.003
Neurological intensive care unit admission (*n* = 91)	19 (20.9%)	11 (20.0%)	8 (22.2%)	0.799
Neurological intensive care unit LOS: Median (IQR)	12 (7–27)	11 (8–13)	20 (6–51)	0.404
Disposition in patients alive on discharge (*n* = 204) Discharged from ED Admitted to and discharged from hospital Died	113 (52.3%) 91 (42.1%) 12 (5.6%)	83 (56.8%) 55 (37.7%) 8 (5.5%)	30 (42.9%) 36 (51.4%) 4 (4.7%)	0.048
Overall LOS for admitted patients (*n* = 91): Median (IQR)	9 (3–21)	11 (4–22)	7 (3–19)	0.148
GOSE at discharge: Median (IQR) 7–8 (lower to upper good recovery) 1–6 (death, vegetative state, or disability) Missing	7 (7–8) 153 (70.8%) 44 (20.4%) 19 (8.8%)	8 (7–8) 110 (75.3%) 26 (17.8%) 10 (6.9%)	7 (6–8) 43 (61.4%) 18 (25.7%) 9 (12.9%)	0.096

^a^ The cumulative percentages for these variables may exceed 100% because the categories within the variable are not mutually exclusive.

AVPU, Alert, Voice, Pain, Unresponsive; CNS, central nervous system; COVID-19, coronavirus disease 2019; CT, computed tomography; ED, Emergency Department; GCS, Glasgow Coma Scale; GOSE, Glasgow Outcome Scale Extended; IQR, interquartile range; LOS, length of stay.

The majority of patients presented with blunt injury (92.6%) and polytrauma (55.1%), with additional injuries most commonly in the extremities (40.7%). Further, 86.6%, 8.8%, and 4.6% of patients presented with mild (GCS score = 13–15), moderate (GCS score = 9–12), and severe (GCS score = 3–8) TBI, respectively. Most patients were conscious (73.6%) on presentation. Sensorimotor deficits were documented among 19% of patients in the ED. Among the 35 patients with spine trauma, incomplete spinal cord injury (SCI) was observed in 42.9% and complete SCI in 14.3%. Neurological decline was observed during care in 11.1% of patients. Computed tomography (CT) imaging was performed for 63.0%, with abnormal imaging findings—including hemorrhage, contusion, or fracture—documented in 33.3%.

The overall mortality rate in the study population was 5.6%. Among the remaining patients, 52.3% of patients were discharged from the ED and 42.1% were discharged from the hospital. The median LOS for hospitalized patients was 9 days (IQR = 3–21 days); 20.9% of patients required neuro-ICU admission, with a median LOS of 12 days (7–27 days); 54.2% of patients received a surgical consultation within 6 h; and 17.6% underwent craniotomy or craniectomy. On discharge, 70.8% had a GOSE score of 7–8 (lower to upper good recovery), whereas 20.4% had a GOSE score of 1–6 (moderate to severe disability, vegetative state, or death).

### Changes in neurotrauma volume, management, and outcomes

**FIG. 2.**
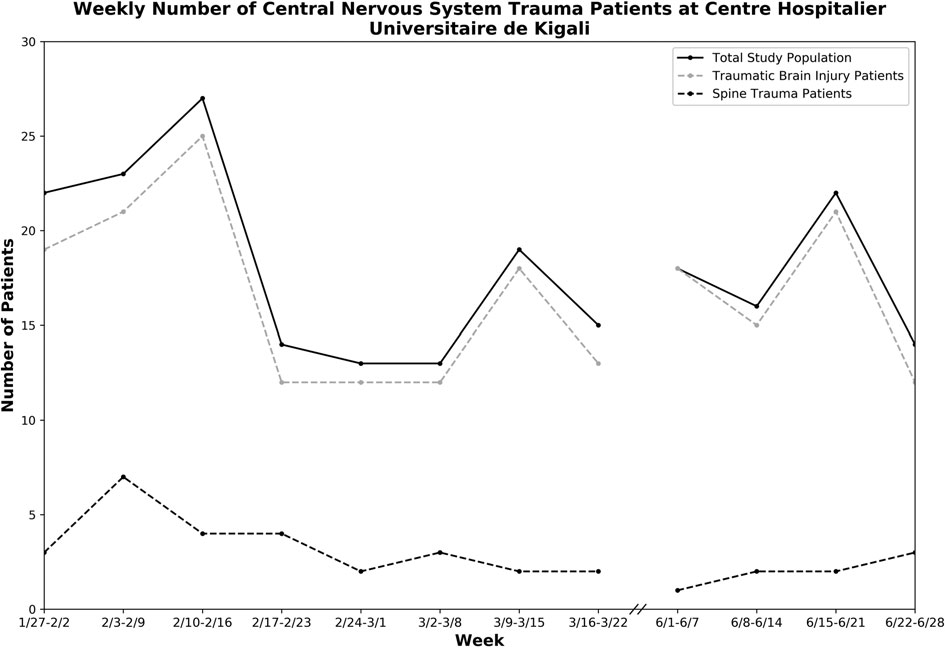
Weekly number of CNS trauma patients at CHUK. Weekly number of CNS trauma patients presenting to the CHUK Emergency Department from January 27, 2020 to June 28, 2020, excluding a cessation of research activities from March 22, 2020 to May 31, 2020 during Rwanda's first national lockdown. Weekly volumes for traumatic brain injury and spine trauma were also individually plotted. CHUK, Centre Hospitalier Universitaire de Kigali; CNS, central nervous system.

Weekly volumes for TBI (mean = 16.5 vs. 17.1, *p* = 0.819) and spine trauma (mean = 2.0 vs. 3.4, *p* = 0.086) patients did not change during the COVID-19 pandemic (Fig. 2). The type of neurotrauma, mechanism of injury, and time from injury to presentation also did not change during this period (Table 1). However, during COVID-19, a higher percentage of treated patients with neurotrauma were male (87.1% vs. 73.3%, *p* = 0.022). A non-significant increase in alcohol use before injury (17.1% vs. 11.0%, *p* = 0.186) and out-of-hospital transfers (48.6% vs. 37.7%, *p* = 0.128) were also observed during the period.

**FIG. 3.**
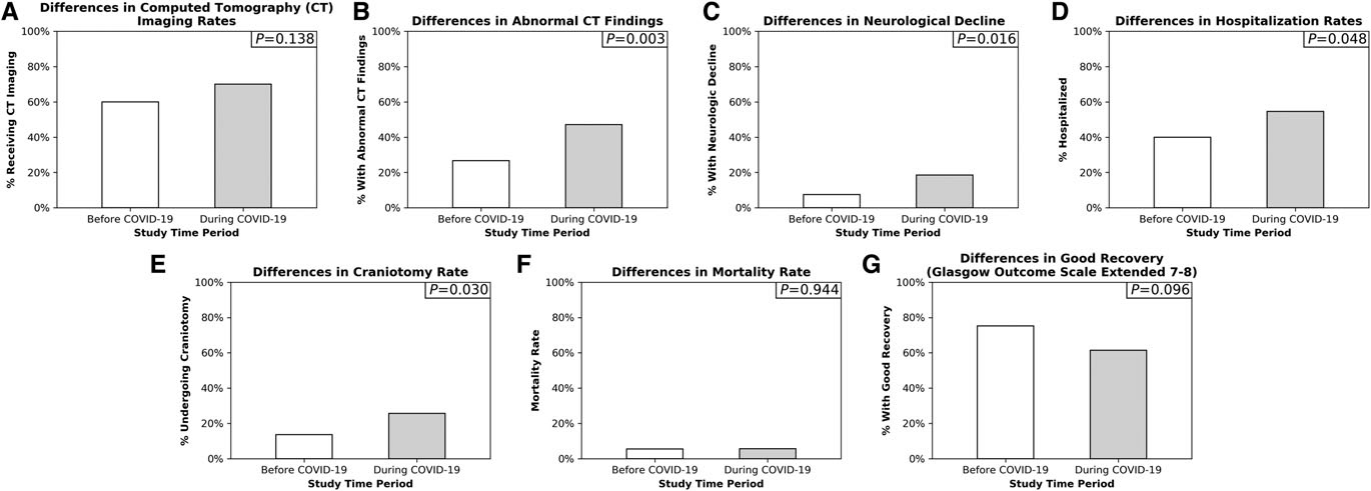
Differences in central nervous system trauma patients before and during COVID-19 pandemic. Differences in trauma presenting severity, management, and outcomes between patients admitted prior to (white) and during (gray) the COVID-19 pandemic. *P*-values for differences between the two time periods were displayed in the top right corner of each panel. **(A)** Difference in CT imaging rates. **(B)** Difference in abnormal CT imaging findings (hemorrhage, contusion, or fracture). **(C)** Difference in neurological decline during treatment (composite of Glasgow Coma Scale decline, new-onset unconsciousness, or new-onset sensorimotor deficit). **(D)** Difference in hospitalization rates. **(E)** Difference in craniotomy rates. **(F)** Difference in mortality rate. **(G)** Difference in Glasgow Outcome Coma Scale Extended 7–8 at discharge (reflecting “good recovery”). COVID-19, coronavirus disease 2019; CT, computed tomography; GCS, Glasgow Coma Scale; GOSE, Glasgow Outcome Coma Scale Extended.

Patients treated during COVID-19 had lower GCS scores (mean = 13.8 vs. 14.3, *p* = 0.068) and KTS (mean = 14.0 vs. 14.3, *p* = 0.097) on ED arrival, both denoting higher injury severity, but these differences only approached significance (Table 1). Although CT imaging rates did not change, patients treated during COVID-19 had higher rates of abnormal imaging findings documented (47.1% vs. 26.7%, *p* = 0.003; Fig. 3). Moreover, although there were no differences in shock index, consciousness, mobility, or sensorimotor deficits on ED presentation, the rates of neurological decline documented during care doubled during COVID-19 (18.6% vs. 7.5%, *p* = 0.016).

During the pandemic, a higher percentage of patients were hospitalized (54.6% vs. 39.9%, *p* = 0.048) rather than discharged from the ED, but overall LOS did not change (*p* = 0.148). Neuro-ICU admission rates and LOS were comparable across study periods. Craniotomy rates increased twofold (25.7% vs. 13.7%, *p* = 0.003) during the pandemic. However, craniotomy rates did not change when only patients with abnormal CT findings (35.9% vs. 51.5%, *p* = 0.182) or neurological decline (61.5% vs. 45.5%, *p* = 0.431) were analyzed. The rates of intubation and administration of antibiotics, antiepileptic drugs, and stress ulcer prophylaxis remained unchanged. Mortality rates (5.5% vs. 5.7%, *p* = 0.944) did not change between study periods. For functional status, a lower percentage of patients were discharged with a GOSE score of 7–8 indicating good recovery (61.4% vs. 75.3%, *p* = 0.096), but this change only approached significance.

## Discussion

Studies have documented changes in the incidence, severity, management, and outcomes of injury patients during the COVID-19 pandemic in HICs, but these trends have been less characterized in LMICs. The present study evaluated differences between patients with CNS trauma treated at CHUK before and during the COVID-19 pandemic. Although weekly volume and mechanisms of injury did not change between study periods, patients with neurotrauma treated during COVID-19 were more likely to be male, present with abnormal CT findings such as hemorrhage or fracture, undergo neurological decline, and require hospitalization and craniotomy procedures. Whereas mortality rates remained unchanged, there was a decrease in the percentage of patients with good functional status on discharge that approached significance. Even during the pandemic, CNS trauma remained an important driver of health care needs and resource utilization, and this study expands the understanding of how management for this clinical entity was impacted and responded to during the pandemic in an LMIC setting.

During COVID-19, Rwanda was the first African country to enact a national lockdown (March 21, 2020) and the nation employed additional strategies including other restrictions on public mobility such as curfews, scaling up testing through pooling, constructing public handwashing stations, and standardizing screening procedures for presenting patients such as utilizing robots for monitoring vital signs.^35–38^ In an analysis by the Lowy Institute, Rwanda was recognized for having the most successful nationwide pandemic response within Africa and the seventh best globally.^23^ Unfortunately, Rwanda was not been unaffected by later waves of the pandemic and briefly reinstated a national lockdown in January-February and July 2021. By the end of 2021, Rwanda had approximately 107,000 confirmed cases of COVID-19 and 1350 deaths.^39^ The success of Rwanda's pandemic response may partially explain why no significant differences during the pandemic were observed for outcomes such as mortality, and the present study's findings may help guide inter-country comparisons to elucidate whether Rwanda's public health measures may have prevented adverse impacts to CNS trauma care delivery during that challenging period.^22^

Prior studies in HICs have demonstrated that caseloads for emergent injuries decreased during the pandemic. Putative drivers for this observed trend include nationwide policies restricting mobility such as social distancing and curfews, as well as decreased and delayed presentation from patients due to concerns of viral transmission.^8–11^ Within Sub-Saharan Africa, these trends have been most studied in South Africa, with earlier research demonstrating a 30–55% decrease in acute trauma patients during similar study periods.^18,21,40–42^ Another study from a tertiary hospital in Nigeria found a 40% drop in this clinical population during the pandemic.^20^

In contrast, this study determined that weekly case volumes for TBI and spine trauma were unchanged following the onset of the pandemic. One explanation for this finding is that this study was unable to assess a 2-month time frame during Rwanda's national lockdown, when research operations were ceased. Another analysis of general injury patients at CHUK, from which the present study population was drawn, found that overall trauma volume indeed dropped threefold during this lockdown period but experienced a significant rise week-by-week, returning to pre-COVID-19 levels by June.^22^ Consequently, it is conceivable that CNS trauma may have experienced similar trends, but the present study could not corroborate this. Another notable trend is that during the pandemic, CHUK experienced a 10.9% increase approaching significance in patients with neurotrauma transferred from outside hospitals. Due to this study population being drawn exclusively from CHUK, it is possible that an intra-COVID decrease in CNS trauma volume within Rwanda as a whole may have been countervailed by an increase in neurotrauma patient transfer to CHUK specifically, resulting in weekly volumes remaining relatively constant.

Moreover, despite restrictions in mobility and confirmation of lower traffic congestion within Rwanda during the pandemic,^43^ mechanisms of injury, including RTA, did not change during Rwanda's first pandemic wave. Although some studies determined that certain mechanisms such as RTA and gunshots decreased during COVID-19 waves, including in LMIC settings,^18,21,44^ other research found no changes.^40,41^ Some studies have hypothesized that despite lower overall traffic, RTA injuries may have remained the same due to increases in risk-taking behavior during this time,^45,46^ which may be reflected by the 6% increase in alcohol use among patients with neurotrauma during the pandemic. The increase in the percentage of male patients with neurotrauma treated at CHUK during COVID-19 has not been found in several earlier assessments of LMIC injury care,^18–21^ and may be due to gender-related differences in risk-taking behaviors or health-seeking, which warrant further study.

In parallel to an earlier assessment of general injury at CHUK, patients with neurotrauma during COVID-19 had higher injury acuity on presentation, such as higher CT imaging abnormalities despite no changes in imaging utilization, and greater rates of hospitalization were required. As discussed earlier, the increase in trauma severity widely documented during COVID-19 may be attributable to decreased willingness of patients with milder injuries to present for care, but the unchanged volumes in this study suggest that this does not sufficiently explain severity differences. However, it is also possible that higher acuity CNS trauma events increased during the pandemic period. Another contributor to higher trauma severity at CHUK may be increased referrals of injury patients with higher case severity from smaller regional centers, due to the burdens of COVID-19 care.^20,22^ In-hospital transfer rates for general injury at CHUK increased significantly during this period, and a 10.9% increase for the neurotrauma population was documented in this study.^22^ Given the specialized nature of neurotrauma care and well-documented shortage of neurosurgeons in Sub-Saharan Africa,^47^ it is important to study these trends to elucidate responses for maintaining the quality of such care, even when burdened with rising in-hospital transfers during pandemic settings.

Finally, rates of neurological decline and performance of neurosurgical intervention doubled for patients with neurotrauma during the pandemic. Higher intervention rates, such as decompressive craniotomy or craniectomy, may have been partially secondary to differences in presenting injury severity, as neurosurgical rates were not significantly different when exclusively looking at patients with abnormal imaging findings or neurological decline. Despite the higher rates of patients requiring hospitalization care, experiencing neurological decline, and undergoing surgery, there was no change in mortality during COVID-19. The same trend was found for general injury patients at CHUK.^22^

As discussed earlier, the lack of increased mortality may be partially due to Rwanda's relative success in addressing COVID-19, which may have reduced burdens in health care worker and resource allocation for COVID-19 care. However, mortality rates do not paint the full picture, as morbidity is another important consequence of neurotrauma, with 8 million DALYs lost annually. In Sub-Saharan Africa, ameliorating post-traumatic morbidity is further challenged by reduced resources to support long-term and rehabilitative care for this population, and studies have accordingly advocated for further evaluation of this outcome in LMIC settings.^48^ In our study, functional status as quantified by the GOSE, a unique end-point analyzed by the present study in comparison with earlier assessments of injury care during COVID-19, showed a non-significant trend toward lower rates of good recovery by discharge during the pandemic. Given the substantive long-term morbidity of neurotrauma,^3,4^ further research into the functional status following discharge of patients with neurotrauma is an important area of further study.

### Limitations

There are limitations to the present study. First, the standardized forms used for prospective data collection were designed with the original intent of evaluating temporal trends associated with acute injury care in the CHUK ED, but the present study represents an adaptation during the COVID-19 pandemic to study intra-period differences in CNS trauma management. As a result, although the present study population was enrolled prospectively, a minority of clinical variables, such as neurological examination findings, had to be collected via retrospective chart review. Second, 77.8% of patients screened for enrollment (668/864) were excluded from analysis. Although the majority of these exclusions were due to not being adult patients with CNS trauma, the exclusion of a high percentage of the general trauma population in CHUK may have introduced bias.

Third, this study only represents selected time periods prior to and during the COVID-19 pandemic, due to following the timeline of a study protocol approved before the onset of the COVID-19 pandemic, and consequently presents results from a limited time frame that may not fully reflect the pandemic's impact on CNS trauma care. Additionally, the influence of seasonal trends could not be accounted for based on the sampling time frames, and due to lack of data on these trends at CHUK, these factors could not be adjusted for. However, the selected study timeline has significance in capturing the institution's early response and adaptations to an unprecedented pandemic situation, and, in the absence of additional information for the context studied, can be used to instruct care and resource allocation for the ongoing pandemic.

Fourth, long-term outcomes following discharge such as 30-day mortality were not tracked and increased study of morbidity following neurotrauma in Sub-Saharan Africa is needed. However, this study was able to evaluate the GOSE as an end-point, which has been validated as a metric for long-term functional status.^30,31^ Fifth, due to CHUK being a single tertiary institution with robust access to injury care and specialty services, these findings may not be broadly generalizable to the country level or other LMICs. Despite these limitations, this study provides some of the only data on CNS-focused trauma care in an LMIC during the COVID-19 pandemic, and it may inform care delivery and public health response to serve this clinical population in under-resourced settings as the pandemic continues.

## Conclusions

At a Rwandan tertiary hospital, neurotrauma volume did not change significantly during the COVID-19 pandemic period evaluated, but presenting patients had higher rates of abnormal CT findings, neurological decline, and craniotomy, with no changes in mortality rates. Characterization of these trends can help inform care and resource allocation during pandemic conditions in Rwanda and other similar LMIC settings.

## Transparency, Rigor, and Reproducibility Statement

This study was not formally registered because study design, variables, and routine clinical care treatment conditions were instead tracked by a Standard Operating Procedures document prepared by all involved institutions before the study began (available on request). The analysis plan was not formally pre-registered, but the team member with primary responsibility for the analysis (lead author) certifies that the analysis plan was pre-specified. A sample size of 200 subjects was planned based on availability of patients with neurotrauma presenting to the study setting during the study timeline. Screening was conducted with 864 potential participants, 216 participated, and adequate data were obtained from 216. Participants were blinded to results of the other assessments throughout the study, even after primary clinical observations were complete. Data collection was performed by investigators blinded to relevant participant characteristics such as predefined group membership (e.g., case vs. control, injury severity category, etc.).

Data analyses were performed by investigators who were aware of relevant characteristics of the participants. Data were acquired between January 27, 2020 and June 28, 2020, excluding a cessation of research activities from March 22, 2020 to May 31, 2020 during Rwanda's first national lockdown. Data were analyzed using Stata (StataCorp, College Station, TX, USA). The time required for data acquisition was 5 months. The time required for pre-processing and analysis was 6 months. All equipment and software used to perform acquisition and analysis are widely available from StataCorp. The key inclusion criteria (e.g., primary diagnosis or prognostic factor) are established standards in the field. Non-parametric statistical tests were used to account for potential non-normality of data. No replication or external validation studies have been performed or are planned/ongoing at this time to our knowledge. De-identified data from this study are available on request. Analytic code from this study are available on request. The authors agree to provide the full content of the manuscript on request by contacting the corresponding author.
